# Dosimetry and optimal scan time of [^18^F]SiTATE-PET/CT in patients with neuroendocrine tumours

**DOI:** 10.1007/s00259-021-05351-x

**Published:** 2021-04-29

**Authors:** Leonie Beyer, Astrid Gosewisch, Simon Lindner, Friederike Völter, Lena M. Mittlmeier, Reinhold Tiling, Matthias Brendel, Clemens C. Cyran, Marcus Unterrainer, Johannes Rübenthaler, Christoph J. Auernhammer, Christine Spitzweg, Guido Böning, F. J. Gildehaus, Klaus Jurkschat, Carmen Wängler, Björn Wängler, Ralf Schirrmacher, Vera Wenter, Andrei Todica, Peter Bartenstein, Harun Ilhan

**Affiliations:** 1 Department of Nuclear Medicine, University Hospital, LMU Munich, Marchioninistraße 15, 81377 Munich, Germany; 2 Department of Radiology, University Hospital, LMU Munich, Munich, Germany; 3 ENETS Centre of Excellence, Interdisciplinary Center of Neuroendocrine Tumours of the GastroEnteroPancreatic System at the University Hospital of Munich (GEPNET-KUM), University Hospital of Munich, Munich, Germany; 4 Department of Internal Medicine 4, University Hospital, LMU Munich, Munich, Germany; 5 Fakultät für Chemie und Chemische Biologie, Technische Universität Dortmund, Dortmund, Germany; 6 Biomedical Chemistry, Department of Clinical Radiology and Nuclear Medicine, Medical Faculty Mannheim of Heidelberg University, Mannheim, Germany; 7 Molecular Imaging and Radiochemistry, Department of Clinical Radiology and Nuclear Medicine, Medical Faculty Mannheim of Heidelberg University, Mannheim, Germany; 8 Department of Oncology, Division of Oncological Imaging, University of Alberta, Edmonton, Alberta Canada

**Keywords:** NET, PET/CT, [^18^F]SiTATE, Dosimetry

## Abstract

**Purpose:**

Radiolabelled somatostatin analogues targeting somatostatin receptors (SSR) are well established for combined positron emission tomography/computer tomography (PET/CT) imaging of neuroendocrine tumours (NET). [^18^F]SiTATE has recently been introduced showing high image quality, promising clinical performance and improved logistics compared to the clinical reference standard ^68^Ga-DOTA-TOC. Here we present the first dosimetry and optimal scan time analysis.

**Methods:**

Eight NET patients received a [^18^F]SiTATE-PET/CT (250 ± 66 MBq) with repeated emission scans (10, 30, 60, 120, 180 min after injection). Biodistribution in normal organs and SSR-positive tumour uptake were assessed. Dosimetry estimates for risk organs were determined using a combined linear-monoexponential model, and by applying ^18^F S-values and reference target masses for the ICRP89 adult male or female (OLINDA 2.0). Tumour-to-background ratios were compared quantitatively and visually between different scan times.

**Results:**

After 1 h, normal organs showed similar tracer uptake with only negligible changes until 3 h post-injection. In contrast, tracer uptake by tumours increased progressively for almost all types of metastases, thus increasing tumour-to-background ratios over time. Dosimetry resulted in a total effective dose of 0.015 ± 0.004 mSv/MBq. Visual evaluation revealed no clinically relevant discrepancies between later scan times, but image quality was rated highest in 60 and 120 min images.

**Conclusion:**

[^18^F]SiTATE-PET/CT in NET shows overall high tumour-to-background ratios from 60 to 180 min after injection and an effective dose comparable to ^68^Ga-labelled alternatives. For clinical use of [^18^F]SiTATE, the best compromise between image quality and tumour-to-background contrast is reached at 120 min, followed by 60 min after injection.

## Introduction

Well-differentiated neuroendocrine tumours (NET) are characterised by an overexpression of somatostatin receptors (SSR) on the cell surface [1] which serve as a target for radiolabelled somatostatin analogues (SSA) used for both diagnostics and therapy (theranostics) [2, 3]. Considering improved detection rates especially in early disease stages, SSR-targeted combined positron emission tomography/computer tomography (PET/CT) contributes to the increasing incidence of this overall heterogeneous group of neoplasms [4]. PET/CT with clinically established ^68^Ga-labelled SSAs is recommended in current guidelines for diagnosis and staging, re-staging, management decisions and monitoring treatment response in neuroendocrine neoplasms [5, 6] and [^68^Ga]Ga-DOTA-TOC/DOTA-TATE has been approved by the FDA and EMA [7].

However, the generator-based approach of ^68^Ga is accompanied by high costs due to limited availability of FDA- and EMA-approved ^68^Ge/^68^Ga generators and low activity amounts after single elution for a maximum of three to four patients per synthesis [8]. The relatively short half-life (68 min) and high positron energy with a maximum of 1.9 MeV lead to a suboptimal spatial resolution, and further impact clinical applicability [9, 10]. Compared to ^68^Ga, production of ^18^F is possible with lower costs for nuclear medicine centres with access to a cyclotron, showing a more practical half-life (110 min) and favourably lower positron energy with a maximum of 635 keV [11, 12]. Therefore, ^18^F-labelled SSA SiTATE (formerly known as ^18^F-SiFA*lin*-TATE) represents a promising alternative for NET PET imaging [13]. The synthesis of [^18^F]SiTATE is based on a one-step ^19^F-^18^F isotopic exchange reaction [14, 15] and has been automated on a Scintomics GRP™ platform [16]. Recently published first in-human data indicated favourable characteristics of [^18^F]SiTATE, i.e. high image quality and significantly higher tracer uptake in most tumour lesions including the liver, lymph nodes and bone metastases [17, 18]. Despite the higher uptake in the liver and spleen, tumour-to-liver (TLR) and tumour-to-spleen ratios (TSR) were proven to be comparable to those of [^68^Ga]Ga-DOTA-TOC and excellent inter-observer agreement between both radioligands underlines the applicability in clinical routine [17].

The aim of this study was to investigate the normal-tissue biodistribution and tumour uptake of [^18^F]SiTATE for different imaging scan times. Furthermore, the radiation exposure of [^18^F]SiTATE-PET/CT was estimated based on longitudinal measurements. For use in clinical routine, quantitative and visual evaluation of tumour delineation served to determine the optimal scan time for PET/CT diagnostics.

## Materials and methods

### Patient enrolment

All patients were referred for imaging by their treating endocrinologists and/or oncologists between February and October 2020 and gave written informed consent to undergo [^18^F]SiTATE-PET/CT following the regulations of the German Pharmaceuticals Act. The study was performed in compliance with the principles of the Declaration of Helsinki and its subsequent amendments [19], and with the approval of the local ethics committee (approval number 20-1077). No patient reported any unforeseen symptoms. No drug-related pharmacologic effects or physiologic responses occurred. Six male and two female patients with differentiated neuroendocrine tumours (G1 *n* = 2, G2 *n* = 5, G3 *n* = 1) and a median age of 68 (range 44–80) presented for [^18^F]SiTATE-PET/CT at our department. Primary tumour locations included the ileum (*n* = 4), pancreas (*n* = 1), lung (*n* = 1) and kidney (*n* = 1); in one patient, no primary tumour was detectable (carcinoma of unknown primary). Most of the patients underwent peptide receptor radionuclide therapy (*n* = 5) and/or surgery (*n* = 4) prior to imaging. Detailed patient characteristics are provided in Table 1.

**Table 1:** Patient characteristics

No.	Sex	Age (y)	Primary tumour	Time since initial diagnosis (m)	Tumour-grade (Ki-67)	Localisation of tumours/metastases	Prior therapies	SSA (at scan time)	Creatinine [mg/dl]/GFR [ml/min] (at scan time)	MBq	Injected peptide mass [μg]
1	M	63	Ileum	120	G1 (2%)	HEP, PER	Surgery, PRRT, SSA	No	1.1/71	272	n.a.
2	M	63	Lung	61	G2 (10%)	OSS	Surgery, RT, CTx, PRRT	Octreotide LAR 30 mg/28 days	1.2/66	220	n.a.
3	F	67	Kidney	248	G2 (5%)	HEP, OSS, LYM	Surgery, PRRT, Xgeva	No	0.9/66	114	1.24
4	F	44	CUP	1	G2 (6%)	LYM	None	No	0.9/80	240	4.19
5	M	72	Ileum	60	G2 (4%)	HEP, LYM	SSA, PRRT	Octreotide LAR 30 mg/28 days	1.0/72	283	1.93
6	M	80	Ileum	153	G2 (5%)	HEP, LYM	SSA, PRRT	Octreotide LAR 30 mg/28 days	1.3/56	316	2.72
7	M	69	Pancreas	16	G2 (15%)	HEP	CTx	No	1.1/71	320	1.82
8	M	76	Ileum	85	G1 (2%)	HEP	Surgery, PRRT, SSA	Octreotide LAR 30 mg/28 days	1.3/53	234	1.77

*M*, male; *F*, female; *CUP*, carcinoma of unknown primary; *HEP*, liver; *PER*, peritoneum; *OSS*, bone; *LYM*, lymph node; *PRRT*, peptide receptor radionuclide therapy ([^177^Lu]Lu-DOTA-TATE); *SSA*, somatostatin analogues; *RTx*, radiotherapy; *CTx*, chemotherapy; *LAR*, long-acting release; *GFR*, glomerular filtration rate; *n.a.*, not available

### PET/CT imaging

SiTATE was obtained from ABX, Advanced Biomedical Compounds (Dresden, Germany), and [^18^F]SiTATE was synthesised as described previously [14, 15]. All quality control data met the release criteria. [^18^F]SiTATE-PET/CT scans were acquired at the Department of Nuclear Medicine, LMU Munich on a Siemens Biograph mCT flow (Siemens Healthineers, Erlangen, Germany). After intravenous injection of 3 ± 1 MBq/kg (mean 250 ± 66 MBq, range 114 to 320, injected peptide mass 2.28 ± 1.05 μg) of [^18^F]SiTATE, PET scans were acquired 10, 30, 60, 120 and 180 min after injection for 15–20 min (in flow mode depending on the body height). Prior to all scans, blood samples were taken from most patients (*n* = 7) and patients were asked to empty the bladder if necessary. In seven patients, contrast-enhanced CT scans with 1.5 mL of iopromide (Ultravist-300, Bayer Healthcare, Leverkusen, Germany) per kilogramme of body weight were performed for anatomic localisation; the remaining case received diagnostic CT scan without contrast enhancement. All patients were asked for discomfort or unusual symptoms after tracer injection and between all scans. The PET scan was acquired by static emission data with a scan speed of 0.7 mm/s for both neck and abdominal region and 0.9 mm/s for the lung region in flow mode. With CT scans serving for attenuation correction, PET images were reconstructed with a transaxial 200 × 200 matrix using TrueX (including TOF, 2 iterations and 21 subsets, 3D Gauss post-filter of 4-mm full width half maximum).

### Image analysis

Image analysis was performed using a dedicated software package (Hermes Hybrid Viewer, Hermes Medical Solutions, Stockholm, Sweden). All metastatic lesions included were identified and measured by the senior author (H.I.). Tumour uptake in patients was assessed by SUV_max_ and SUV_mean_ (threshold 50% of max) measurements for all scan times as described previously [17]. To assess the biodistribution of normal organs, spherical VOIs were placed inside the organ parenchyma using a 1-cm-diameter VOI for small organs (thyroid, parotid gland, myocardium, adrenal glands) and a 2-cm-diameter VOI for muscle, liver, spleen, kidney, fat tissue, aortic lumen (descending aorta), lung, bone (femur), uterus, prostate, pancreas (tail), small intestine and colon. Because of the high variation in volume, the urinary bladder content was measured by placing a VOI around the whole urinary bladder, separately for each single image, with volume correction. Tumour-to-liver ratios (TLR) and tumour-to-spleen ratios (TSR) were calculated for the most frequent tumour lesion types (bone, liver, lymph nodes, peritoneal) according to the clinically relevant Krenning score which has been evaluated for SSTR-PET imaging [20, 21].

### Radiation dosimetry estimate

Patient-related time activity information was gathered during the biodistribution analysis. The time-integrated activity was assessed by using a combined linear-monoexponential model; i.e. linear interpolation of time activity data points was performed until the maximum uptake, followed by a monoexponential fit to the subsequent data points to describe the time activity curve (TAC) post-maximum. Regarding the description of the blood TAC, a bi-exponential model was employed. S-values for ^18^F were based on the ICRP89 reference adult male and female (OLINDA 2.0) [22]. The effective dose was determined using ICRP103 tissue weighting factors (OLINDA 2.0). Regarding the total bone marrow absorbed dose, the cross-absorbed dose from biodistribution organs and the remainder of the body was considered, as well as the absorbed dose from blood activity circulation. The latter employed the patient haematocrit to derive the red marrow-to-blood activity concentration ratio [23]. All dosimetry results are reported with respect to ICRP89 reference adult male or female organ masses.

### Visual analysis

Images of later scan times (60, 120, 180 min p.i.) with higher tumour-to-background ratios compared to earlier scan times (10, 30 min p.i.) were compared visually by six readers (three less experienced resident nuclear medicine physicians with an average of 2.3 years of experience in SSA-PET: LB, LM, FV; three more experienced senior nuclear medicine physicians with an average of 10.7 years of SSA-PET experience: RT, AT, MU) blinded to the acquisition starting point (to guarantee an objective, uninfluenced rating), assessing the image quality (1 excellent, 2 good, 3 moderate, 4 poor, 5 non-diagnostic) and amount of detectable metastases (0 non-metastatic, 1 uni = 1, 2 oligo = 2–5, 3 multi > 5/disseminated). The readers had access to clinical background information and received series numbers of the different scan times in random order. The images were rated separately and not in conjunction. For both the less experienced and more experienced readers, the majority read was used for illustration of results.

### Statistical analysis

Data are reported as mean or median ± standard deviation as stated. Tumour-to-background ratios (tumour-to-liver SUV_mean_, tumour-to-spleen SUV_mean_) were compared between scan times using a one-way analysis of variance. Image quality ratings between different scan times were compared using a Friedman test for multiple comparisons between both less and more experienced readers. GraphPad Prism (version 8.4.3, GraphPad Software Inc., San Diego, CA, USA) was used for statistical analysis and illustration of results. A significance level of *p* < 0.05 was applied in all analyses.

## Results

### Biodistribution and dosimetry estimate

**Fig. 1:**
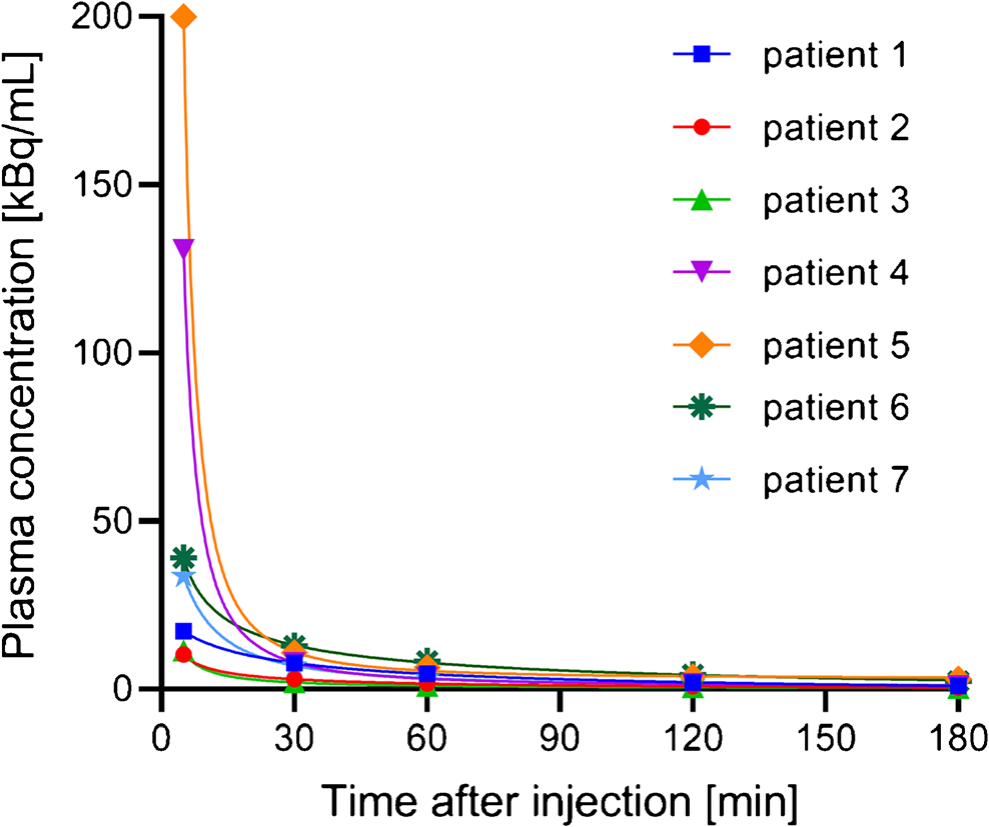
Blood activity curves from time of injection to 3 h after injection. Different colours represent different patients

[^18^F]SiTATE showed a fast blood pool washout over time. Mean counts per millilitre decreased by 78 ± 13% from 10 to 30 min and by 87 ± 8% from 10 to 60 min after injection. In one patient, blood sampling was not possible due to difficult venous access. For blood activity curves of all remaining patients, see Fig. 1.

**Fig. 2:**
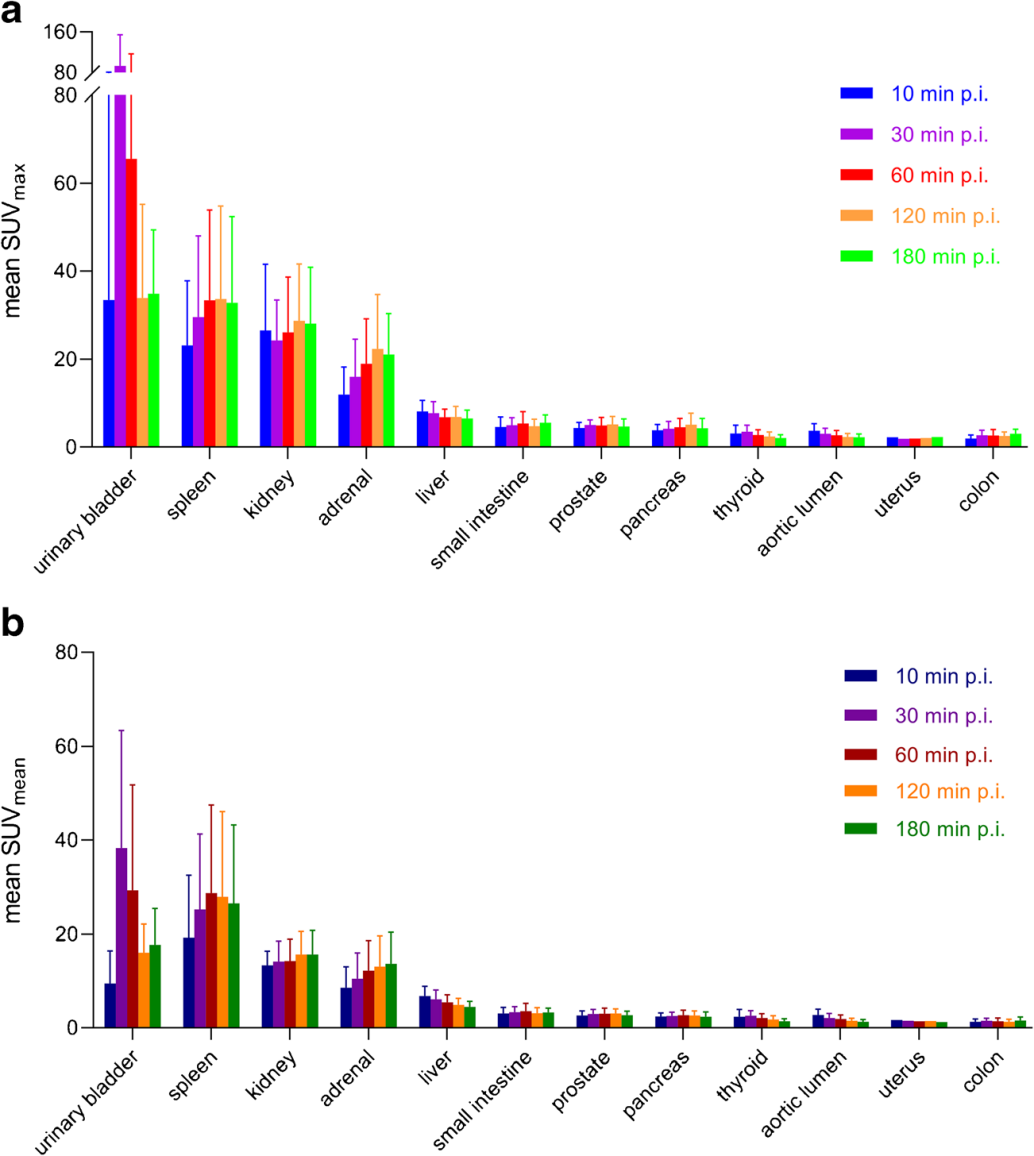
Biodistribution of [^18^F]SiTATE over time for acquisition starting points 10, 30, 60, 120 and 180 min after injection for **a** SUV_max_ and **b** SUV_mean_ values of normal organs with highest radiotracer accumulation. SUV, standard uptake value; p.i., post-injection. Error bars represent standard deviations

The normal organs showed a constant tracer uptake after 1 h with only minimal changes between 1 and 3 h post-injection. The highest SUV_max_ and SUV_mean_ were noted in the urinary bladder, the spleen, the kidneys and the adrenal glands, followed by the liver, small intestine, prostate (*n* = 6), pancreas, thyroid, aortic lumen, uterus (*n* = 1) and colon. Figure 2 shows the biodistribution in all normal organs over time. Low SUV_max_ (< 2.0) and SUV_mean_ (< 1.4) values were observed in the myocardium, parotid gland, bone, muscle, lung and fat tissue (data not shown in the graph).

The approximated dosimetry for all patients together is presented in Table 2. The effective dose of [^18^F]SiTATE was 0.015 ± 0.004 mSv/MBq for all patients (0.022 ± 0.006/0.014 ± 0.003 mSv/Bq for female/male patients), resulting in an effective dose of 3.8 mSv for a mean injected activity of 250 MBq.

**Table 2:** Dosimetry estimates (OLINDA) for [^18^F]SiTATE and the ICRP89 reference adult organ masses

Site	Absorbed organ dose (mSv/MBq)		
	Female *n* = 2	Male *n* = 6	All *n* = 8
Adrenal glands	0.130	0.076	0.087
Brain	0.007	0.006	0.006
Breasts	0.009	–	0.009
Oesophagus	0.015	0.010	0.011
Eyes	0.007	0.006	0.006
Gallbladder wall	0.024	0.018	0.018
Lower large intestine wall	0.017	0.012	0.013
Small intestine	0.028	0.018	0.020
Stomach	0.023	0.012	0.015
Upper large intestine wall	0.021	0.015	0.016
Rectum	0.013	0.010	0.011
Heart wall	0.019	0.016	0.015
Kidneys	0.129	0.095	0.100
Liver	0.071	0.040	0.044
Lungs	0.015	0.011	0.011
Ovaries	0.013	–	0.013
Pancreas	0.043	0.026	0.028
Salivary glands	0.015	0.024	0.012
Prostate	–	0.012	0.012
Red marrow	0.016	0.011	0.012
Bone surface	0.010	0.007	0.008
Spleen	0.294	0.107	0.144
Testes	–	0.007	0.007
Thymus	0.011	0.008	0.008
Thyroid	0.023	0.014	0.015
Urinary bladder wall	0.034	0.046	0.042
Uterus	0.013	–	0.013
Effective dose (mSv/MBq)	0.022 ± 0.006	0.014 ± 0.003	0.015 ± 0.004

### Tumour uptake and tumour-to-background ratios

**Fig. 3:**
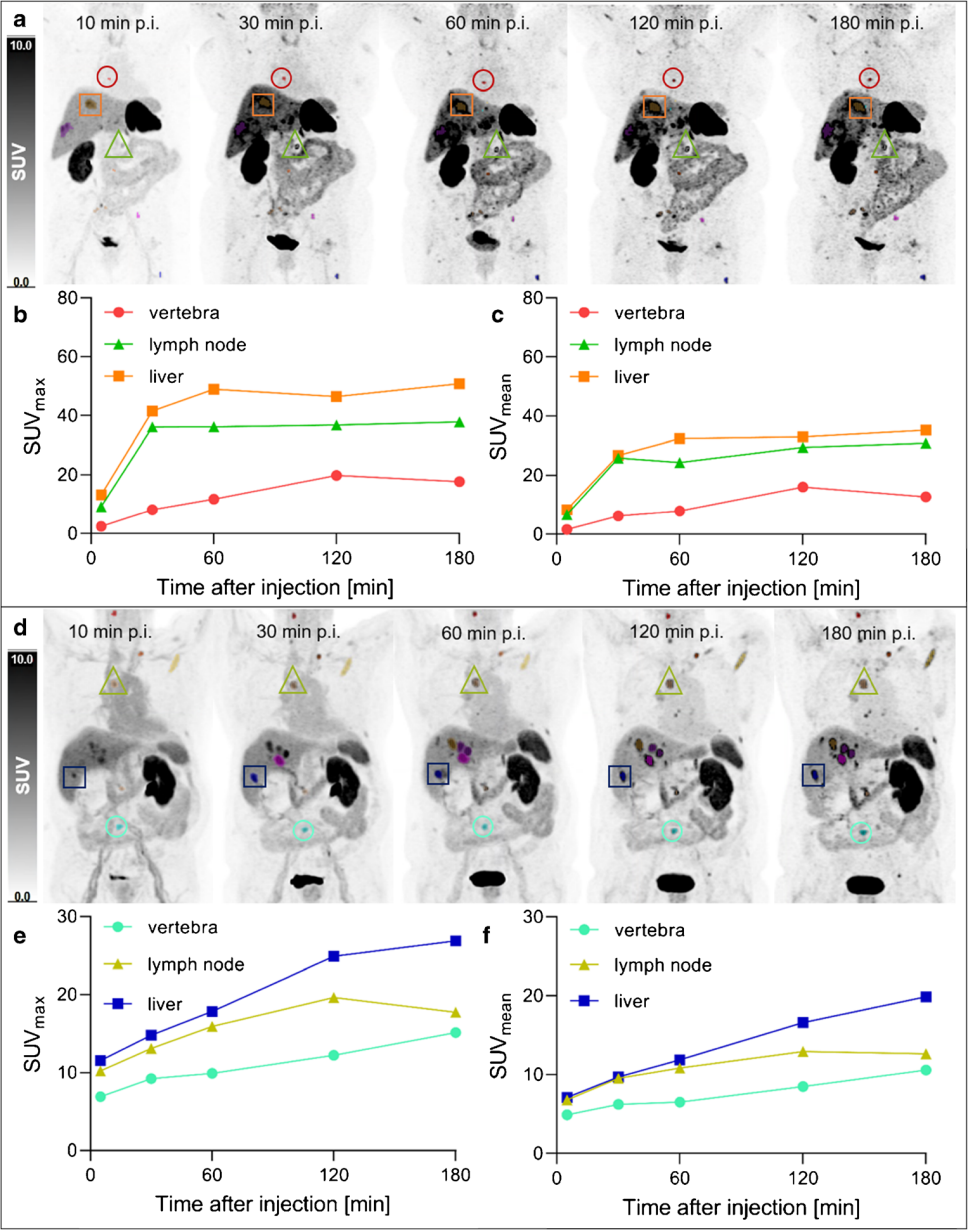
**a**, **d** Exemplary maximum intensity projection images from all scan times (10, 30, 60, 120, 180 min p.i.) in two exemplary patient cases (upper image: patient 3, female, 114 MBq [^18^F]SiTATE, no SSA medication, creatinine 0.9 mg/dl, GFR 66 ml/min; bottom image: patient 6, male, 316 MBq [^18^F]SiTATE, SSA medication, creatinine 1.3 mg/dl, GFR 56 ml/min) with corresponding time activity curves of three exemplary metastatic lesions (vertebra, lymph node, liver) from **b, e** SUV_max_ and **c, f** SUV_mean_ values. p.i., post-injection; SUV, standardised uptake value; SSA, somatostatin analogues; GFR, glomerular filtration rate

A total of 68 metastatic lesions (bone *n* = 41, liver *n* = 15, lymph node *n* = 8, peritoneal *n* = 4) were assessed in all patients. Images (maximum intensity projections) from all scan times and corresponding activity curves of three exemplary metastases (bone, liver, lymph node) in two exemplary patient cases are shown in Fig. 3.

**Fig. 4:**
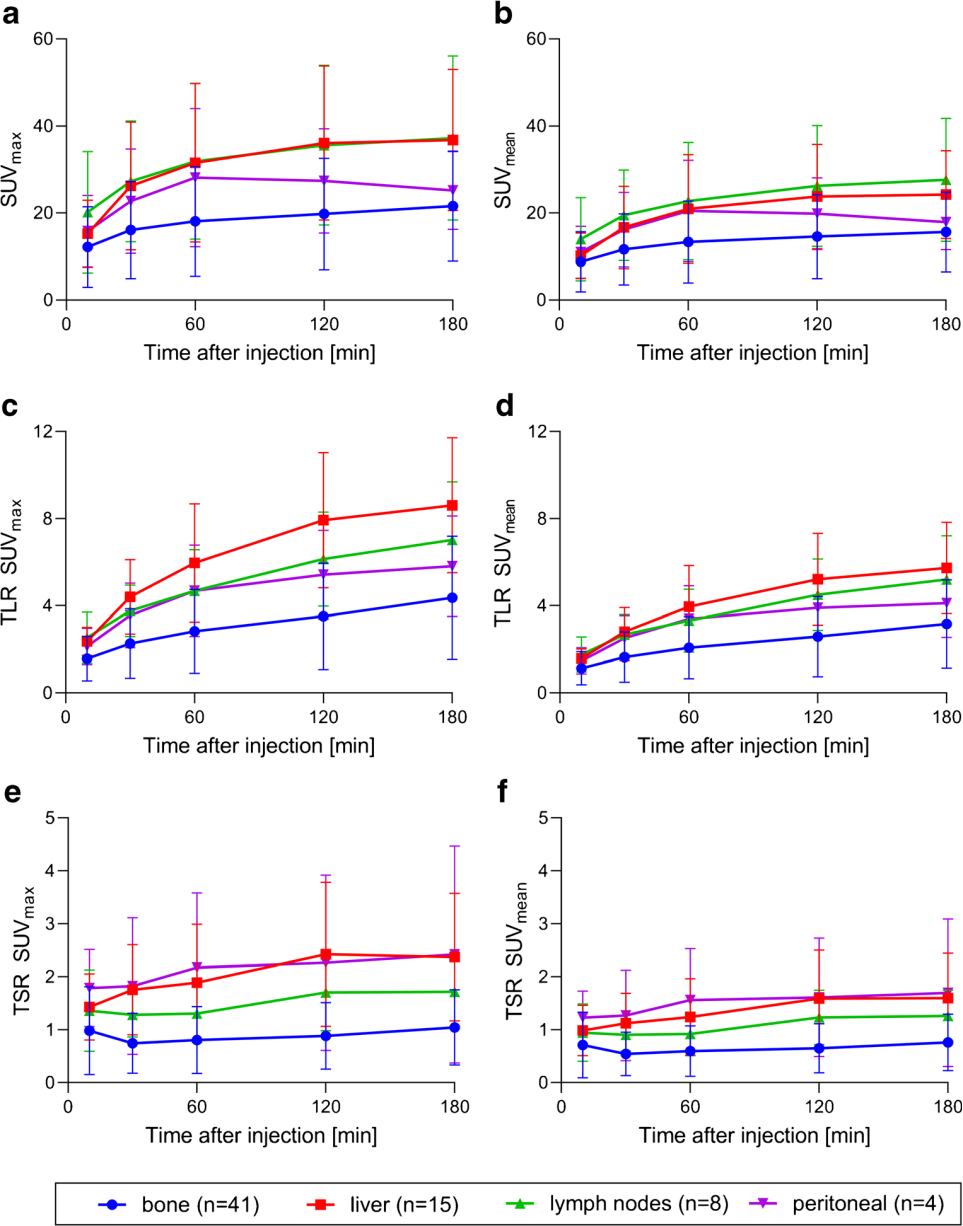
Tumour uptake values (**a** SUV_max_ and **b** SUV_mean_) and tumour-to-background ratios (**c** TLR SUV_max_, **d** TLR SUV_mean_, **e** TSR SUV_max_, **f** TSR SUV_mean_) for all types of metastatic lesions (bone, liver, lymph nodes, peritoneal) and different scan times. SUV, standardised uptake value; TLR, tumour-to-liver; TSR, tumour-to-spleen

Tumour uptake increased for all types of metastases from time of injection to 60 min after injection, and further increased for almost all types of metastases (expect peritoneal lesions) until 180 min after injection. Comparing the different scan times, tumour-to-background ratios were significantly higher for later scan times for both TLR and TSR (SUV_mean_ ratios TLR 60 min vs. 120 min: 2.7 ± 1.7 vs. 3.5 ± 2.1, + 28%, *p* < 0.001; 60 min vs. 180 min: 2.7 ± 1.7 vs. 4.0 ± 2.3, + 48%, *p* < 0.001; 120 min vs. 180 min: 3.5 ± 2.1 vs. 4.0 ± 2.3, + 16%, *p* < 0.001; SUV_mean_ ratios TSR 60 min vs. 120 min: 0.8 ± 0.6 vs. 1.0 ± 0.8, + 18%, *p* < 0.001; 60 min vs. 180 min: 0.8 ± 0.6 vs. 1.1 ± 0.8, + 27%, *p* < 0.001, 120 min vs. 180 min: 1.0 ± 0.8 vs. 1.1 ± 0.8, + 8%, *p* = 0.010). Absolute tumour uptake values and TLR/TSR ratios for all scan times are illustrated for different metastatic lesion types in Fig. 4.

### Visual evaluation of different scan times

**Fig. 5:**
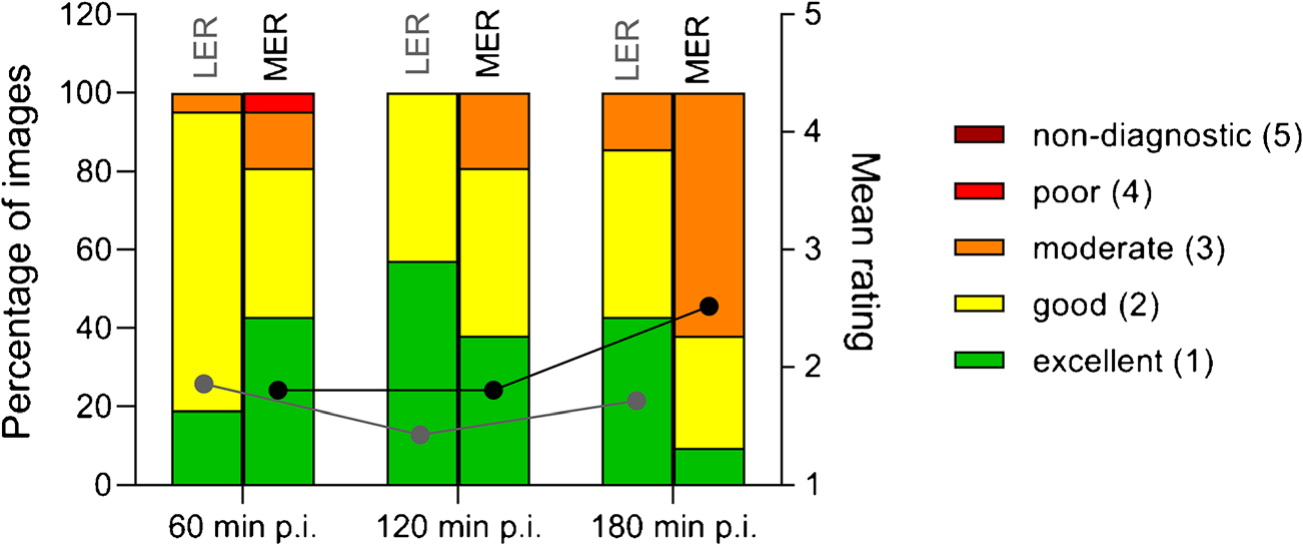
Image quality ratings of acquisition starting points 60, 120 and 180 min after injection. The lines with dots display average ratings separately for LER (grey line) and MER (black line). LER, less experienced readers; MER, more experienced readers; p.i., post-injection

Image quality was rated excellent or good in the majority (80%) of cases. Images acquired after 60 and 120 min p.i. did not show significantly different ratings for both less (*p* = 0.35) and more experienced readers (*p* = 1.00). For less experienced readers, the highest score was reached for 120 min p.i. images with 1.4 ± 0.5, followed by 180 min p.i. with 1.7 ± 0.7 and 60 min p.i. with 1.9 ± 0.5, but differences were not statistically significant (60 vs. 120 min: *p* = 0.347, 120 vs. 180 min: *p* > 0.999). The more experienced readers equally preferred the 60 min p.i. (1.8 ± 0.9) and 120 min p.i. (1.8 ± 0.7) images; 180 min p.i. images were rated worse (2.5 ± 0.7) when compared to both 60 min p.i. (*p* = 0.04) and 120 min p.i. (*p* = 0.03) images. For a visualisation of image quality ratings, see Fig. 5.

All images were also rated according to the amount of metastatic tumour lesions (bone, liver, lymph node, peritoneal) for all patients. Of all rated images and tumour lesions (0 ratings), no discrepancies were found between 60 and 120 min p.i. images. Between those two and 180 min p.i. images, only two discrepancies were found for less experienced readers (patient no. 2: oligo-metastatic vs. multi-metastatic in liver metastasis and patient no. 7: uni-metastatic vs. oligo-metastatic in bone metastases), whereas no deviations were found for more experienced readers.

**Fig. 6:**
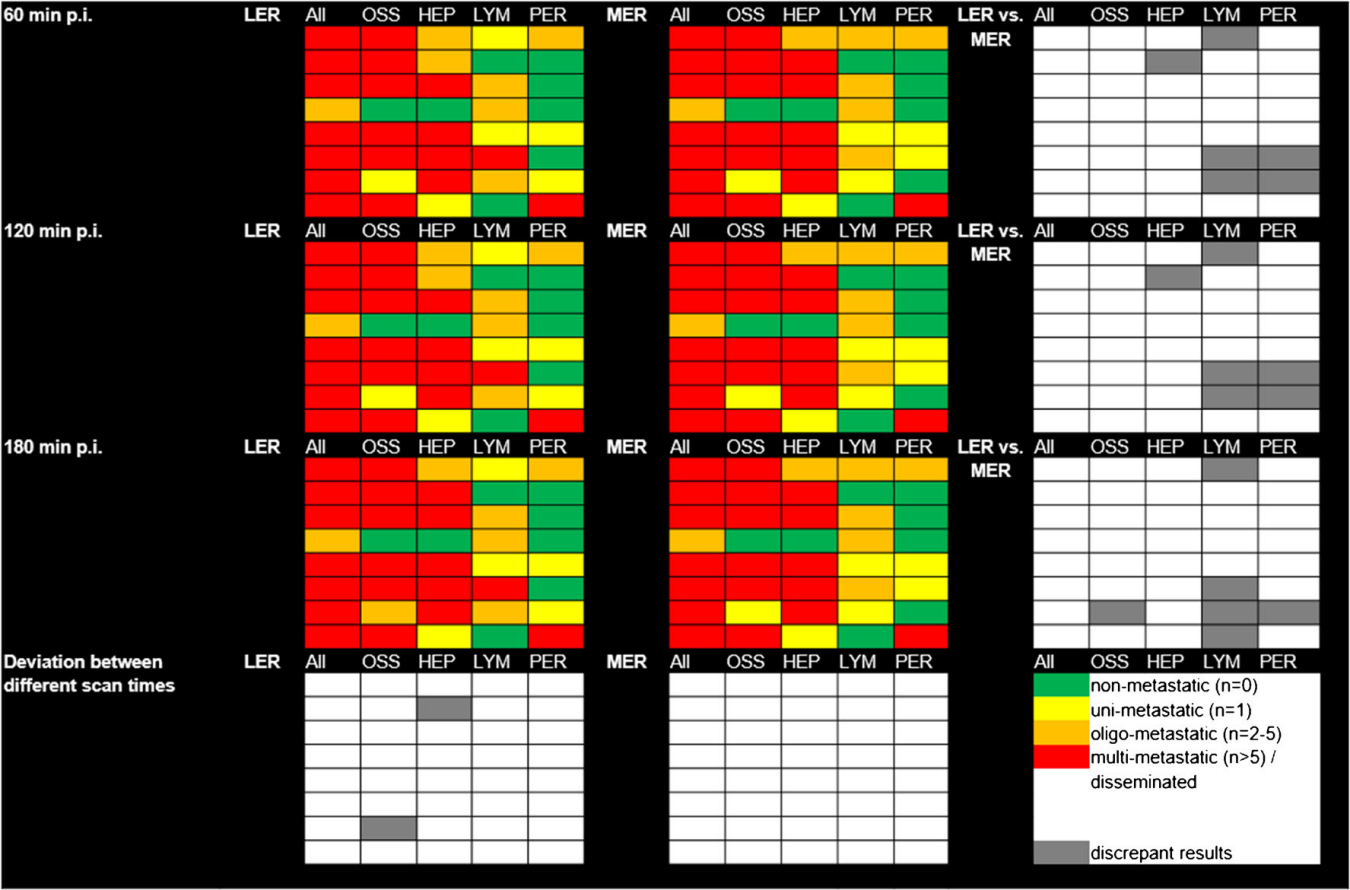
Visual rating of all metastatic tumour lesions. Each row displays the rating in one patient with the deviation between different scan times in the bottom row. Different columns represent ratings (majority read) for less experienced (LER) and more experienced (MER) readers with the deviation between both in the rightmost column. OSS, bone metastases; HEP, liver metastases; LYM, lymph node metastases; PER, peritoneal metastases; p.i., post-injection

Comparing less and more experienced readers, discrepancies occurred equally frequently for all scan times (6/40). Figure 6 comprises all rating results separately for all patients. As stated, the majority read is displayed for both less and more experienced readers.

## Discussion

[^18^F]SiTATE recently emerged as a promising ^18^F-labelled SSR-targeting peptide, challenging the ^68^Ga-labelled clinical reference standards for imaging of NET [11, 24] as recommended in the current guidelines [5, 6]. As described previously, radiosynthesis was proven to be feasible and efficient [16, 17]. For a broad application in clinical practice, further data on tracer kinetics is necessary to estimate dosimetry and to determine the optimal scan time for PET/CT imaging with [^18^F]SiTATE. In the present study, we provided first dosimetry data of [^18^F]SiTATE imaging in eight NET patients indicating long-lasting tracer uptake and excellent tumour-to-background ratios for later scan times. Furthermore, the radiation exposure was slightly lower compared to ^68^Ga-labelled alternatives.

Considering amongst others the favourable logistics and radiosynthesis as well as the prolonged half-life of ^18^F, radiotracer development aims to provide ^18^F-labelled alternatives for most important PET imaging targets [11]. The direct comparison between ^68^Ga- and ^18^F-labelled radiopharmaceuticals targeting the prostate-specific membrane antigen (PSMA) has already provided first evidence of a slightly lower radiation exposure caused by ^18^F-labelled compounds when compared to ^68^Ga (0.017/0.013/0.011 vs. 0.021 mSv/MBq) [25–28]. In line with this, the effective dose for [^18^F]SiTATE was 0.015 ± 0.004, whereas former studies reported 0.021–0.026 mSv/MBq for [^68^Ga]Ga-DOTA-TOC, [^68^Ga]Ga-DOTA-TATE and the newly developed compound [^68^Ga]Ga-DOTA-JR11 [29–31]. The biodistribution and absorbed dose in normal organs was comparable to [^68^Ga]Ga-DOTA-TOC and [^68^Ga]Ga-DOTA-TATE, with the urinary bladder and spleen being exposed to the highest radiotracer uptake followed by the kidneys and adrenal glands [30].

As already shown during the patient-wise comparison between [^18^F]SiTATE and [^68^Ga]Ga-DOTA-TOC, tumour-to-background ratios did not significantly differ for most tumour lesions and broadly indicated sufficient contrast when imaged 60 min after tracer injection [17]. This could be confirmed via the longitudinal measurements presented in this study. Whereas tracer uptake in normal organs remained stable from 60 to 180 min after injection, tumour uptake further increased for almost all types of metastases (except peritoneal lesions), leading to even higher tumour-to-background ratios for later scan times. In contrast to those findings, longitudinal measurements of [^68^Ga]Ga-DOTA-JR11 in examples of a cohort of 20 patients showed a constant decrease of tracer uptake in liver metastasis post-peak between 20 and 40 min p.i. [31].

A higher tumour-to-background contrast for later scan times raises the question of the optimal acquisition starting point. It has already been shown for other radiotracers that later scan times potentially lower uptake in the blood pool and normal organs and thereby increase the tumour-to-background contrast [32]. Nevertheless, a longer waiting period between tracer injection and PET/CT acquisition is accompanied by complicated patient logistics with respect to clinical routine and significantly longer waiting times. Therefore, a diagnostic benefit of later acquisitions must be demonstrated. Despite the fact that the highest tumour-to-background contrasts were observed for 180 min p.i., image quality was rated significantly worse by experienced readers compared to 60 and 120 min p.i. images, probably due to the lower count statistics and consequently increased noise. Interestingly, less experienced readers were not misguided by the higher noise 180 min p.i., potentially because higher contrasts increased their diagnostic confidence and thereby had a positive influence on their image quality rating. Overall, no clinical relevant discrepancies could be displayed between different scan times for the detection of metastatic lesions (between visually evaluated scan times 60, 120 and 180 min p.i.). Comparing 60 min p.i. and 120 min p.i. images, quality was rated equal by the more experienced readers and slightly better at 120 min p.i. by the less experienced readers. However, evaluation of all metastatic sites between these two scan times was identical separately for both less and more experienced readers. Although 120 min p.i. seems to be the optimum regarding image quality, the obtained results suggest that imaging 60 min p.i. will not result in a decreased detection rate. We will implement a modified clinical protocol that schedules an image acquisition start approximately 90 min after injection, which we regard as the best compromise between optimum image quality and logistical considerations. Similar results have been reported for [^18^F]Al-1,4,7-triazacyclononane-1,4,7-tri-acetate-octreotide ([^18^F]AlF-OC), a SSR-targeting imaging agent synthetised using the chelator-based Al^18^F-method [33, 34], with increasing TBR for liver, bone and lymph node metastases at scan times from 60 to 180 min p.i. Comparable to our study, the detection rate was not influenced by increasing TBR over time [33].

Based on dosimetry data, the mean radiation exposure of 3.8 mSv for 250 MBq [^18^F]SiTATE in our cohort is in agreement with the range of former studies dealing with [^68^Ga]Ga-DOTA-TOC or [^68^Ga]Ga-DOTA-TATE (2.1–4.8 mSv) [29–31], although the effective dose per administered activity implies a slightly lower exposure for [^18^F]SiTATE. This can be explained by higher injected activities used in this study (250 ± 66 MBq [^18^F]SiTATE) compared to the reference studies (91 ± 19 MBq [^68^Ga]Ga-DOTA-TOC [30], 87 ± 16 MBq/185 MBq [^68^Ga]Ga-DOTA-TATE [29, 30], 185 ± 2 MBq [^68^Ga]Ga-DOTA-JR11 [31]). As illustrated in Fig. 3, which compares imaging results for the patients who received the lowest (114 MBq) and nearly the highest (316 MBq) activities, the high tumour-to-background contrast characteristic of [^18^F]SiTATE leads to a sufficient image quality also for lower activities (1.4 MBq/kg for this example). These findings suggest that lower administered activities between 100 and 120 MBq (1.3–1.5 MBq/kg for 80 kg) might be sufficient (resulting in an average radiation exposure of 1.7 mSv), but studies with higher patient numbers are needed to confirm this assumption.

The major limitations of this study are the small sample size and heterogeneity of our study cohort with a wide range of injected activities due to the clinical routine setting. We included patients with different neuroendocrine tumour subtypes (not only gastroenteropancreatic NET) for a representative patient selection from the clinical routine and transferability for all different types of tumour entities. Considering a possible tumour sink effect on healthy organ dosimetry [35], a higher number of patients would be favourable. Furthermore, considering the half-life of ^18^F, later acquisition times (e.g. additional scans at 4 and 5 h p.i.) would have been favourable for dosimetry. However, as patients included in these analyses all suffer from metastatic disease with reduced general health condition, only a small subset will tolerate longitudinal measurements over a total period of 3.5 h.

## Conclusions

Compared to clinical reference standards, [^18^F]SiTATE shows slightly lower radiation exposure and high tumour-to-background ratios in different metastatic lesion types, increasing over time. For use in clinical practice, the best imaging strategy as a compromise between image quality and tumour-to-background contrast is reached 120 min after injection, with 60 min p.i. as a close second.

## Data Availability

The data that support the findings of this article are available from the corresponding author (H.I.) upon reasonable request.

## References

[1] Reubi J, Waser B, Schaer JC, Laissue JA (2001). Erratum to: Somatostatin receptor sst1-sst5 expression in normal and neoplastic human tissues using receptor autoradiography with subtype-selective ligands. Eur J Nucl Med.

[2] Zidan L, Iravani A, Kong G, Akhurst T, Michael M, Hicks RJ. Theranostic implications of molecular imaging phenotype of well-differentiated pulmonary carcinoid based on (68)Ga-DOTATATE PET/CT and (18)F-FDG PET/CT. Eur J Nucl Med Mol Imaging. 2020. 10.1007/s00259-020-04915-7. 10.1007/s00259-020-04915-7 32572559

[3] Werner RA, Weich A, Kircher M, Solnes LB, Javadi MS, Higuchi T (2018). The theranostic promise for neuroendocrine tumors in the late 2010s - where do we stand, where do we go?. Theranostics..

[4] Dasari A, Shen C, Halperin D, Zhao B, Zhou S, Xu Y (2017). Trends in the incidence, prevalence, and survival outcomes in patients with neuroendocrine tumors in the United States. JAMA Oncol.

[5] Bozkurt MF, Virgolini I, Balogova S, Beheshti M, Rubello D, Decristoforo C (2017). Guideline for PET/CT imaging of neuroendocrine neoplasms with (68)Ga-DOTA-conjugated somatostatin receptor targeting peptides and (18)F-DOPA. Eur J Nucl Med Mol Imaging.

[6] Sundin A, Arnold R, Baudin E, Cwikla JB, Eriksson B, Fanti S (2017). ENETS consensus guidelines for the standards of care in neuroendocrine tumors: radiological, nuclear medicine & hybrid imaging. Neuroendocrinology.

[7] Hennrich U, Benešová M. [(68)Ga]Ga-DOTA-TOC: the first FDA-approved (68)Ga-radiopharmaceutical for PET imaging. Pharmaceuticals (Basel, Switzerland). 2020;13. 10.3390/ph13030038. 10.3390/ph13030038 PMC7151717 32138377

[8] Banerjee SR, Pomper MG (2013). Clinical applications of Gallium-68. Appl Radiat Isot.

[9] Conti M, Eriksson L (2016). Physics of pure and non-pure positron emitters for PET: a review and a discussion. EJNMMI Phys.

[10] Kemerink GJ, Visser MG, Franssen R, Beijer E, Zamburlini M, Halders SG (2011). Effect of the positron range of 18F, 68Ga and 124I on PET/CT in lung-equivalent materials. Eur J Nucl Med Mol Imaging.

[11] Sahnoun S, Conen P, Mottaghy FM (2020). The battle on time, money and precision: Da[(18)F] id vs. [(68)Ga]liath. Eur J Nucl Med Mol Imaging.

[12] Cole EL, Stewart MN, Littich R, Hoareau R, Scott PJ (2014). Radiosyntheses using fluorine-18: the art and science of late stage fluorination. Curr Top Med Chem.

[13] Niedermoser S, Chin J, Wängler C, Kostikov A, Bernard-Gauthier V, Vogler N (2015). In vivo evaluation of ^18^F-SiFAlin-modified TATE: a potential challenge for ^68^Ga-DOTATATE, the clinical gold standard for somatostatin receptor imaging with PET. J Nucl Med.

[14] Wängler C, Waser B, Alke A, Iovkova L, Buchholz HG, Niedermoser S (2010). One-step ^18^F-labeling of carbohydrate-conjugated octreotate-derivatives containing a silicon-fluoride-acceptor (SiFA): in vitro and in vivo evaluation as tumor imaging agents for positron emission tomography (PET). Bioconjug Chem.

[15] Schirrmacher R, Bradtmöller G, Schirrmacher E, Thews O, Tillmanns J, Siessmeier T (2006). 18F-labeling of peptides by means of an organosilicon-based fluoride acceptor. Angew Chem Int Ed Eng.

[16] Lindner S, Simmet M, Gildehaus FJ, Jurkschat K, Wängler C, Wängler B (2020). Automated production of [(18)F]SiTATE on a Scintomics GRP™ platform for PET/CT imaging of neuroendocrine tumors. Nucl Med Biol.

[17] Ilhan H, Lindner S, Todica A, Cyran CC, Tiling R, Auernhammer CJ (2020). Biodistribution and first clinical results of (18)F-SiFAlin-TATE PET: a novel (18)F-labeled somatostatin analog for imaging of neuroendocrine tumors. Eur J Nucl Med Mol Imaging.

[18] Ilhan H, Todica A, Lindner S, Boening G, Gosewisch A, Wängler C (2019). First-in-human (18)F-SiFAlin-TATE PET/CT for NET imaging and theranostics. Eur J Nucl Med Mol Imaging.

[19] World Medical Association Declaration of Helsinki: ethical principles for medical research involving human subjects. Journal international de bioethique = International journal of bioethics. 2004;15:124–9. 15835069

[20] Hope TA, Calais J, Zhang L, Dieckmann W, Millo C (2019). (111)In-pentetreotide scintigraphy versus (68)Ga-DOTATATE PET: impact on Krenning scores and effect of tumor burden. J Nucl Med.

[21] Krenning EP, Valkema R, Kooij PP, Breeman WA, Bakker WH, deHerder WW (1999). Scintigraphy and radionuclide therapy with [indium-111-labelled-diethyl triamine penta-acetic acid-D-Phe1]-octreotide. Ital J Gastroenterol Hepatol.

[22] Stabin MG, Sparks RB, Crowe E (2005). OLINDA/EXM: the second-generation personal computer software for internal dose assessment in nuclear medicine. J Nucl Med.

[23] Hindorf C, Glatting G, Chiesa C, Lindén O, Flux G (2010). EANM Dosimetry committee guidelines for bone marrow and whole-body dosimetry. Eur J Nucl Med Mol Imaging.

[24] Waldmann CM, Stuparu AD, van Dam RM, Slavik R (2019). The search for an alternative to [(68)Ga]Ga-DOTA-TATE in neuroendocrine tumor theranostics: current state of (18)F-labeled somatostatin analog development. Theranostics..

[25] Plyku D, Mena E, Rowe SP, Lodge MA, Szabo Z, Cho SY (2018). Combined model-based and patient-specific dosimetry for (18)F-DCFPyL, a PSMA-targeted PET agent. Eur J Nucl Med Mol Imaging.

[26] Piron S, De Man K, Van Laeken N, D’Asseler Y, Bacher K, Kersemans K (2019). Radiation dosimetry and biodistribution of (18)F-PSMA-11 for PET imaging of prostate cancer. J Nucl Med.

[27] Hohberg M, Kobe C, Krapf P, Täger P, Hammes J, Dietlein F (2019). Biodistribution and radiation dosimetry of [(18)F]-JK-PSMA-7 as a novel prostate-specific membrane antigen-specific ligand for PET/CT imaging of prostate cancer. EJNMMI Res.

[28] Hofman MS, Eu P, Jackson P, Hong E, Binns D, Iravani A (2018). Cold kit for prostate-specific membrane antigen (PSMA) PET imaging: phase 1 study of (68)Ga-tris (hydroxypyridinone)-PSMA PET/CT in patients with prostate cancer. J Nucl Med.

[29] Walker RC, Smith GT, Liu E, Moore B, Clanton J, Stabin M (2013). Measured human dosimetry of 68Ga-DOTATATE. J Nucl Med.

[30] Sandström M, Velikyan I, Garske-Román U, Sörensen J, Eriksson B, Granberg D (2013). Comparative biodistribution and radiation dosimetry of 68Ga-DOTATOC and 68Ga-DOTATATE in patients with neuroendocrine tumors. J Nucl Med.

[31] Krebs S, Pandit-Taskar N, Reidy D, Beattie BJ, Lyashchenko SK, Lewis JS (2019). Biodistribution and radiation dose estimates for (68)Ga-DOTA-JR11 in patients with metastatic neuroendocrine tumors. Eur J Nucl Med Mol Imaging.

[32] Badawi RD, Shi H, Hu P, Chen S, Xu T, Price PM (2019). First human imaging studies with the EXPLORER total-body PET scanner. J Nucl Med.

[33] Pauwels E, Cleeren F, Tshibangu T, Koole M, Serdons K, Dekervel J (2020). [(18)F]AlF-NOTA-octreotide PET imaging: biodistribution, dosimetry and first comparison with [(68)Ga]Ga-DOTATATE in neuroendocrine tumour patients. Eur J Nucl Med Mol Imaging.

[34] Tshibangu T, Cawthorne C, Serdons K, Pauwels E, Gsell W, Bormans G (2020). Automated GMP compliant production of [(18)F]AlF-NOTA-octreotide. EJNMMI Radiopharm Chem.

[35] Beauregard JM, Hofman MS, Kong G, Hicks RJ (2012). The tumour sink effect on the biodistribution of 68Ga-DOTA-octreotate: implications for peptide receptor radionuclide therapy. Eur J Nucl Med Mol Imaging.

